# Ischemic Time as a Predictor of Physical Recovery in the First Months after Heart Transplantation

**DOI:** 10.5402/2012/907102

**Published:** 2012-06-18

**Authors:** Francisco Buendía-Fuentes, Luis Almenar-Bonet, Luis Martínez-Dolz, Ignacio Sánchez-Lázaro, María Rodríguez-Serrano, Diana Domingo-Valero, María José Sancho-Tello de Carranza, Antonio Salvador-Sanz

**Affiliations:** ^1^Heart Failure and Transplant Unit, La Fe University Hospital, Boulevar Sur S/N, 46026 Valencia, Spain; ^2^Cardiology Department, La Fe University Hospital, Boulevar Sur S/N, 46026 Valencia, Spain; ^3^Research Institute, La Fe University Hospital, Boulevar Sur S/N, 46026 Valencia, Spain; ^4^Arrhythmology and Electrophysiology Department, La Fe University Hospital, Boulevar Sur S/N, 46026 Valencia, Spain

## Abstract

Functional results after heart transplantation range from modest to spectacular improvement. Little is known about factors to predict functional result. This study aimed to identify these factors. We present a prospective study including all consecutive transplant recipients (*n* = 55) in a two-year period whose survival was greater than two months. Perioperative, donor, and recipient issues were systematically analyzed. Exercise capacity was assessed by symptom-limited treadmill exercise testing two months after transplantation. Exercise capacity was classified as satisfactory or poor depending on achieving or not 4.5 METs (metabolic equivalents), respectively. Thirty-three patients (60%) showed a good exercise capacity (>4.5 METs), whereas the remaining twenty-two patients (40%) were unable to exceed this threshold. The variables which correlated with exercise capacity in univariate analysis were recipient age, inotropic treatment, ischemic time, ventricular assist device, etiology, urgent transplant, and INTERMACS score. Among them only recipient age and ischemic time were proved to be correlated with exercise capacity in the multiple regression analysis. Thus, younger patients and those who had received an organ with shorter ischemic time showed greater exercise capacity after transplant. These findings strengthen the trend toward reducing ischemic time as much as possible to improve both survival and clinical recovery.

## 1. Introduction

 Heart transplantation (HT) has been shown to increase survival in selected patients with advanced heart failure. Beside survival, transplantation also greatly improves quality of life and exercise capacity [1, 2], although transplanted persons continue to have a lower exercise capacity than healthy persons [3]. In this context, various studies have tried to analyze possible variables related to exercise capacity after transplantation [1–6]. Cardiac, pulmonary, and muscular factors as well as immunosuppressive regimen and exercise training have been linked to exercise intolerance. However, the factors limiting exercise performance during posttransplantation period remain unclear, and little is known about which parameters could predict maximal exercise capacity in cardiac transplant recipients. Studies focus on identifying clinical features predictive of posttransplant exercise [7–10], agree on the importance of recipient age and heart rate response during exercise to predict greater exercise capacity, but differ on the importance of other factors such as donor age, gender, and recipient body mass index [8–10]. Few of these studies analyzed exercise capacity in the first months, even though this parameter adds information on patients' capacity of recovery after transplant. The present study aimed to prospectively analyze the influence of certain donor, recipient, and surgical factors on exercise capacity at two months after heart transplantation.

## 2. Patients and Methods

 A prospective study was designed including all consecutive patients (*n* = 69) undergoing HT at our center in a two-year period, from April 2008 to April 2010. Heart-lung transplants (*n* = 1), retransplants (*n* = 1), followup at another center (*n* = 1), survival less than two months (*n* = 9), and reluctance to sign the consent form (*n* = 2) were excluded. A total of 55 patients were finally included. 

 The study was approved by the ethics committee of our hospital. All included patients gave their written consent to participate in the study.

 Donor, recipient, and perioperative variables were prospectively studied (Table 1). Patient status before HT was estimated by the INTERMACS score [11, 12] and the NYHA (New York Heart Association) scores. We considered urgent transplant when the patient was listed as UNOS (United Network for Organ Sharing) status I. We also took into account the need of using ventricular assist device (extracorporeal membrane oxygenation cardiopulmonary support) previous to heart transplantation.

Organ ischemic time was defined as the time interval from application of donor aortic cross-clamp to release of recipient cross-clamp.

Study patients received immunosuppressive medication guided by side effects and regular histological monitoring of right ventricular endomyocardial biopsies following the same pattern in all cases. Induction therapy was daclizumab at a dose of 1 mg/kg on days 1 and 15. Patients received three daily doses of intravenous methylprednisolone (10 mg/kg) during the first three days after transplant and then received oral deflazacort tapered gradually to the minimum dose necessary to prevent rejection. Cyclosporine or tacrolimus was initiated after transplant when hemodynamics and creatinine had stabilized, usually between 3 and 10 days. Target serum cyclosporine and tacrolimus levels were 200–300 ng/mL and 10–15 ng/mL, respectively, during the first six months after HT. Mycophenolate mofetil (1000 mg BID) was also given from the second day after the transplant. In case of neutropenia or gastrointestinal complications, mycophenolate mofetil was reduced to 500 mg/8 h.

According to the study protocol, we considered it as a rejection when antirejection therapy was given following either clinical features or endomyocardial biopsy results.

Two months after transplantation, patients performed a treadmill symptoms-limited exercise test (General Electric T-2100 Treadmill) according to the Bruce protocol. Hemodynamic parameters (heart rate and blood pressure) were measured at rest, during exercise, and during recovery from exercise. Exercise capacity was estimated by the number of METs (metabolic equivalents) achieved. The exercise test was stopped whenever requested by the patient for dyspnea, weakness, dizziness, pain, or any other reason. All exercise tests were performed by the same cardiologist who was unaware of any donor clinical or surgical data.

To facilitate statistical analysis, the variable exercise capacity was dichotomized by classifying patients into two categories (high or low exercise capacity) based on whether or not its value exceeded 4.5 METs, as reported in the literature [13, 14].

**Figure 1:**
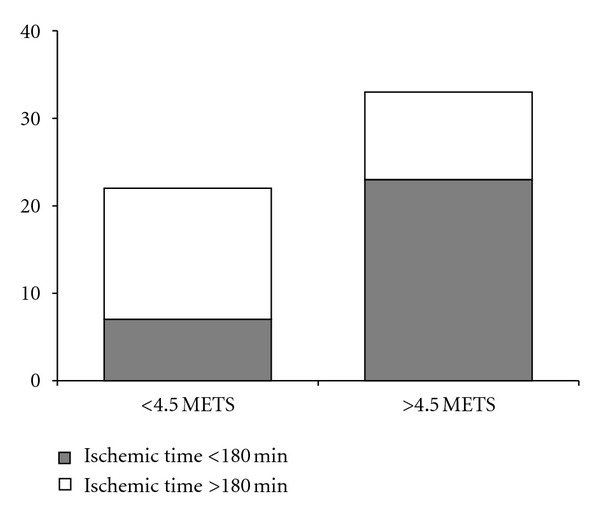
Distribution of ischemic time (> or <180 min) in both groups depending on achieving or not 4.5 METs (metabolic equivalents) in the effort test.

The variable ischemic time was analyzed as a continuous variable, although we made a division between ischemic time greater or less than 180 min, based on the literature [15], to represent results in Figure 1. A variable was also included in the analysis on whether BMI (body mass index) matching between the donor and recipient (donor BMI/recipient BMI) was inside or outside of the range usually considered acceptable in transplant protocols (0.8–1.2) [15].

A preliminary analysis of the relationship between the study variables and exercise capacity was done by univariate analysis (*χ* ^2^ test for qualitative variables and Students *t*-test for quantitative ones). The variables achieving a value of *P* < 0.1 in the univariate analysis were then included in enter multivariate analysis model. 

 ROC curve analysis was also performed in order to highlight the utility of ischemic time to predict effort capacity.

 Variables such as heart rate or maximum heart rate capacity were not included in the analysis because they are directly related to exercise time and therefore to exercise capacity adding little information about anticipating exercise intolerance.

 Calculations were performed with the SPSS 15.0 statistical program, and statistical significance was set at *P* < 0.05.

## 3. Results

The clinical profile of the patients analyzed is shown in Table 1. The bicaval technique was used in all cases. Exercise tests were performed at 62.35 ± 15.4 days from transplant. Thirty-three patients (60%) exceeded 4.5 METs, whereas the remaining twenty-two patients (40%) were unable to exceed this exercise threshold. The variables which correlated with exercise capacity (*P* < 0.1) in the univariate analysis were recipient age (*P* = 0.008), need of inotropic treatment (*P* = 0.086), ischemic time (*P* = 0.004), need of ventricular assist device (*P* = 0.011), etiology (*P* = 0.027), urgent transplant (*P* = 0.057), and INTERMACS score (*P* = 0.026).

 Of the 33 patients who exceeded 4.5 METs, 23 (69.7%) had an ischemic time < 180 min. In contrast, of the 22 patients with poorer exercise capacity, 15 (68.2%) had an ischemic time > 180 min (Figure 1).

**Figure 2:**
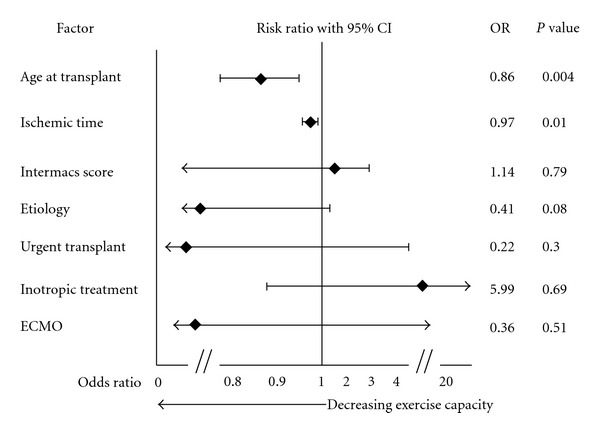
Predictors of exercise capacity included in the multivariate model. Recipient age and ischemic time reached *P* < 0.05. They both were inversely related to exercise capacity.

The multivariate analysis including the seven variables listed above, which reached *P* < 0.1, showed that recipient age and organ ischemia time were inversely related to exercise capacity at 60 days of transplant (Figure 2). There was no significant difference in the rest of variables.

**Figure 3:**
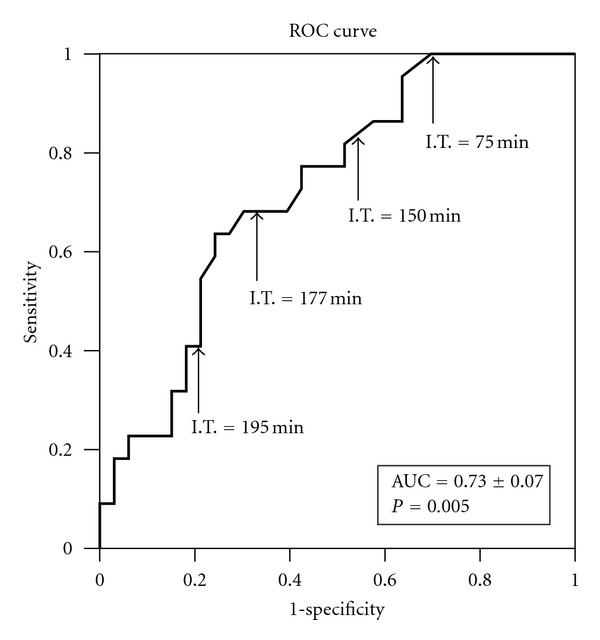
ROC curve for ischemic time values and development of exercise capacity >4.5 METs (metabolic equivalents) two months after heart transplant. The cut-off value considered optimal for classifying the patients was 177.5 min. With this value, we achieved a sensitivity of 73% and a specificity of 58%. The area under the curve (AUC) was 0.73 ± 0.07.

 Moreover, ischemic time ROC curve (Figure 3) showed a good capacity to predict effort capacity (Figure 3), with an AUC (area under the curve) = 0.73 ± 0.07 (*P* = 0.005). The best cutoff was 177.5 min of cold ischemia with a sensitivity = 73% and specificity = 58%.

## 4. Discussion

In addition to the increase in survival resulting from heart transplantation in patients with advanced heart failure, we should not underestimate the improvement in functional status and quality of life achieved with this therapeutic option.

Various studies have focused on clarifying the clinical variables that may influence patient recovery after transplant and more specifically on improvement of the patient's physical capacity [1, 3]. Aside from the variables obtained in the exercise test itself [9], only recipient and donor age, recipient gender, and BMI have been related to exercise capacity [7, 8, 10]. The studies conducted in this regard do not usually include patients in the first months after transplant, although it would be useful to identify some variable relating exercise capacity to physical recovery of the patients in the first stage after transplant. This study included the most important clinical and therapeutic variables that, a priori, could be related to patients' capacity of physical recovery after transplant. The results show that, beside recipient age, only ischemic time was related to exercise capacity at two months from transplant. According to our results, each minute of cold ischemia would increase the odds of not passing 4.5 METs in the effort test. Other important factors such as INTERMACS score, need of inotropic treatment, etiology, need of ventricular assist device, and urgent transplant correlate with effort capacity only in univariate analysis.

 Previous studies have shown an increase in mortality with more prolonged ischemic time [16, 17], especially when combined with older donor age [18, 19]. Although these data have not been confirmed in longer-term followups [20], the current attitude is to try to reduce ischemic time as much as possible. It has therefore been proposed that the optimal time limit for ischemia is below 180 minutes [15]. The results of this study provide further support for the importance of minimizing ischemia time, by relating this parameter for the first time with patient exercise capacity in the first months after transplant.

 One of the study limitations, in addition to the number of patients, is the fact that the exercise test was done without a gas exchange study. However, we consider that quantification of METs as a measure of physical capacity is an equally valid method given that its direct relationship to oxygen consumption (1 MET = 3.5 mL/min/kg resting oxygen consumption) is more achievable in daily clinical practice and does not introduce greater bias because it was quantified under the same conditions in all patients. Another limitation could be the fact of not including the total steroid dose. All patients followed the same immunosuppressive therapy, and there was no difference in rejection episodes; therefore the total steroid dose should be similar in both groups.

## 5. Conclusion

Among all of the recipient and donor clinical variables and the surgical and peri-heart-transplant treatment characteristics, only recipient age and shorter ischemic time were correlated with better exercise capacity at two months of transplant.

The results of this study ratify the importance of minimizing organ ischemic time in heart transplantation not only to reduce mortality but also to improve the exercise capacity of these patients.

## Figures and Tables

**Table 1:** Clinical characteristics in both groups depending on achieving or not 4.5 METs (metabolic equivalents) in the effort test.

	<4.5 METs	>4.5 METs
	*n* = 22	*n* = 33
Days after surgery	59.1 ± 11.4	63.8 ± 17.3
Recipient age (years)^∗^	56.1 ± 9.3	48.9 ± 9.7
Donor age (years)	39.4 ± 11.6	42.4 ± 11.1
Male recipients	68.2	75.7
Male donors	68.2	57.6
Donor BMI/recipient BMI	1 ± 0.17	0.99 ± 0.19
Recipient BMI	25.1 ± 3.7	24.6 ± 3.5
PCP (mmHg)	24.5 ± 10.9	23.7 ± 10.3
PVR (WU)	2.4 ± 1.8	2.3 ± 1.4
LVEF (%)	19.1 ± 11.3	18.7 ± 10.2
Creatinine (mg/dl)	1.1 ± 0.5	1.1 ± 0.4
Ischemic time (min)^∗^	190.9 ± 45.9	151.3 ± 48.8
Ischemic time > 180 min^∗^	68.2	30.3
Inotropic treatment^#^	45.5	22.7
NYHA ≥ III-IV	81.8	78.8
INTERMACS score^∗^	3.6 ± 1.3	4.4 ± 1.3
Diabetes mellitus	27.3	15.2
Hypertension requiring treatment	31.8	27.3
Baseline heart disease (ischemic/idiopathic/hypertrophic/other)^∗^	59/18/0/23	33.3/45.5/12.1/9.1
Smoking <1 years	36.4	27.3
Ventricular assist device^∗^	31.8	6.1
Urgent transplant^#^	45.4	21.2
Moderate-severe obstructive respiratory pattern	13.7	15.5
Treated rejection episodes	31.8	27.3

Values are expressed as mean ± standard deviation or as percentages.

Body mass index (BMI), pulmonary capillary pressure (PCP), pulmonary vascular resistance (PVR), left ventricular ejection fraction (LVEF), New York Heart Association (NYHA), Wood unit (WU) *P < 0.05, ^#^ *P* < 0.1.
